# Gender Differences in Eating Disorder Risk among NCAA Division I Cross Country and Track Student-Athletes

**DOI:** 10.1155/2019/5035871

**Published:** 2019-02-03

**Authors:** Paul A. Krebs, Christopher R. Dennison, Lisa Kellar, Jeff Lucas

**Affiliations:** ^1^Premier Health, Department of Family Medicine, Wright State University Boonshoft School of Medicine, 2261 Philadelphia Drive, Dayton, Ohio 45406, USA; ^2^Department of Sociology, University at Buffalo, SUNY, USA; ^3^Department of Family Medicine, Wright State University Boonshoft School of Medicine, USA; ^4^Department of Health and Sport Science, University of Dayton, USA

## Abstract

**Purpose:**

This study compared gender differences in eating disorder risk among NCAA Division I cross country and track distance running student-athletes.

**Methods:**

Six hundred thirty-eight male and female student-athletes competing at distances of 800m or greater completed the Eating Disorder Screen for Primary Care (ESP). Scores on the ESP were used as the risk of eating disorders.

**Results:**

Females screened positive at higher rates for risk of eating disorders than males on the ESP at a cutoff of 2 (sensitivity 90-100%, specificity 71%) with rates of 45.95% ± 3.03 and 13.66% ± 1.80, respectively. Females were also screened positive at higher rates than males at a stricter cutoff of 3 (sensitivity 81%, specificity 92%), with rates of 21.69% ± 2.50 compared to 4.64% ± 1.10, respectively.

**Conclusion:**

This study highlights that, among distance runners, both males and females are at risk of eating disorders, with females being at higher risk. It also emphasizes the need for screening for risk of eating disorders in this population.

## 1. Introduction

Research on the topic of eating disorders in athletes has shown mixed results. For instance, some studies show no increased risk of eating disorders [1, 2], whereas others suggest a significant increase in the prevalence of these issues in athletes compared to the general population [3, 4]. Certain sports, namely, endurance sports like distance running, have been observed to have higher rates of eating disorders compared to other sports [4, 5]. Research suggests this is due to the sociocultural pressures within the sport, as well as the emphasis on leanness for perceived improvements in performance [6, 7]. Unfortunately, achieving this ‘ideal physique' can potentially drive athletes towards heavy training loads and restrictive diets, which inevitably place them at higher risk for eating disorders [8].

Among female distance runners in particular, eating disorders have been associated with low self-esteem and other mental health conditions [6]. Population studies have shown higher rates of eating disorders in females compared to males, both within the general population and amongst athletes [4, 9]. Thus, it would be reasonable to assume that female distance runners have a higher risk of eating disorders than males in the same sport. However, most studies have not focused on a sport-specific population. Further, it would seem that the emphasis on a lean physique and perceived performance improvements would also place male athletes participating in these sports at increased risk of eating disorders compared to the general population. Few studies, however, have examined this question [10, 11]. With this in mind, male athletes with eating disorders may be underrepresented both in the literature and in practice [12, 13]. In order to adequately identify and treat both male and female athletes with eating disorders, it is important to understand the impact eating disorders have on these populations, particularly in subsets of the athletic population believed to be more vulnerable [12]. This study examines the risk of eating disorders in Division I cross country and track distance running athletes, and examines gender differences in screening results.

## 2. Methods

Data used in this study were collected from “The Collegiate Distance Running Survey,” a cross-sectional study conducted in 2016 using an online survey completed by NCAA Division I distance running student-athletes. A link to an on-line, anonymous survey was forwarded to Division I track and cross country coaches in 2016, and coaches were then able to decide whether or not to forward the survey to their team. After attempting to contact all coaches from the 312 universities sponsoring women's track and Field and 272 universities sponsoring men's track and field in 2016, coaches from twenty-five universities responded with confirmation that they had forwarded the survey to their teams.

Informed consent was obtained prior to participants completing the survey. Participants in this survey competed in NCAA Division I cross country and/or track, at distances equal to or greater than 800 meters. The survey was deemed exempt under the Federal criteria 45 CFR 46.101(b)(2) by the Institutional Review Board (IRB) at Wright State University. Six hundred thirty-eight (638) respondents completed survey questions related to eating disorders and were included in the study.

To screen for eating disorders, student-athletes' responses (1=yes, 0=no) to the Eating Disorder Screen for Primary Care (ESP) were compared. This screen includes the following questions: are you dissatisfied with your eating patterns; do you ever eat in secret; does your weight affect the way you feel about yourself; do you currently suffer with or have you ever suffered in the past with an eating disorder. The ESP was considered a practical substitute for the Questionnaire for Eating Disorder Diagnosis (Q-EDD) and other screening or diagnostic questionnaires for a few reasons. First, its length (i.e., 4 questions for the ESP versus 50 questions for the Q-EDD), sensitivity (90-100%), and specificity (71%) suggest that it is a robust, parsimonious screen for eating disorders [14]. The ESP has also been previously used in university settings (similar to those solicited for this survey) as well as primary care settings [14, 15]. Furthermore, when compared to similar length validated studies for eating disorders, the ESP has been found to have a higher sensitivity while maintaining a similar specificity [14, 16, 17]. When compared to longer surveys such as the Eating Disorder Diagnostic Scale (EDDS), the ESP performed well in screening for eating disorders, as well [15]. Additionally, the ESP questions have been thought to be less invasive, which may allow participants to be more honest in their responses and feel more comfortable while completing the survey [16]. Finally, in the “International Olympic Committee (IOC) Consensus Statement on Periodic Health Evaluation of Elite Athletes,” the ESP was noted as one of the possible screening tools for risk of eating disorders that can be used when screening elite athletes [18]. Taken together, these facts show that the ESP gives a reasonable, clinically relevant estimate of the risk of eating disorders in the population under investigation.

Using the ESP questions, we created dichotomous measures indicating whether respondents screened negatively (i.e., answered affirmatively on 1 or fewer items) or positively (i.e., answered affirmatively on 2 or more items). We also parsed out responses to the ESP with measures indicating rates at which respondents answered affirmatively to 0, 1, 2, or 3-4 of the questions, respectively. These additional tests of robustness were conducted since increasing the cutoff for a positive screen from 2 to 3 on the ESP has been shown to increase the specificity to 92%, although sensitivity decreases to 81% [14]. Finally, we included measures indicating a respondent's age and body mass index (*BMI*).

## 3. Results

Table 1 shows the percentages and standard errors for each ESP question between males and females, as well as the average count of positive responses. All significance tests are based on results from two-tailed, independent samples Z-tests and T-Tests. Of the 638 collegiate distance runners participating in the survey, 57% were male (i.e., 366 respondents) and 43% were female (i.e., 272 respondents). Overall, a higher percentage of females answered positively to the ESP questions. For instance, 30.51% of females answered positively to the questions, “are you dissatisfied with your eating patterns,” whereas 18.85% of males answered positively. The largest disparity between males and females was observed in the question about how one's weight affects their feelings, as 69.49% of females answered positively, compared to 28.69% of males. With regard to the average count of positive responses (with a possible range of 0 to 4), the average count was .62 for males. This means that males typically responded positively to zero, or at most, 1 ESP question. Females, however, had an average count of 1.55. This means that females typically responded positively to 1 or 2 of the ESP questions.

To examine differences in the risk of positive screens for eating disorders between men and women, Table 2 reports the percentages of respondents who reported 0 or 1 positive response to the ESP questions (i.e., a negative screen), as well as the percentages of respondents who reported 2 (or more than 2) positive responses to the ESP questions (i.e., a positive screen).

Indeed, a significantly higher percentage of female athletes were at risk of a positive screen for eating disorders compared to male athletes. That is, 13.66% of male distance runners answered positively to at least 2 ESP questions, whereas 45.95% of female athletes answered positively to at least 2 ESP questions. When examining the disparities in the risk of a positive screen among those who answered positively to at least 3 ESP questions, 4.65% of males were at risk for a positive screen, whereas 21.69% of females were at risk. All told, the higher risk of screening positive for eating disorders among female athletes compared to male athletes was robust with multiple screening criteria. Yet, a notable proportion of male distance running athletes—a subset of the athletic population often ignored in these types of studies—reported symptoms of eating disorders.

## 4. Discussion

This study examined differences in eating disorder risk between male and female distance running athletes. Overall, results were consistent with prior research, as we observed a higher rate of eating disorder risk in females compared to males [4, 9, 10]. In fact, three times as many females screened positive compared to males, with 46% of females screening positive and 14% of males screening positive. Patterns were robust when employing a more specific threshold of 3 or more positive responses to ESP questions. This ratio of 46% of females screening positive compared to 14% of males is similar to ratio seen by Hilbert in the general population, where 5.9% of females showed eating disorder psychopathology compared to 1.5% of men [10], and to studies looking at athletic populations. A 2013 study with elite adolescent athletes showed 14% of females compared to 3% of males had eating disorders [3], and Sundgot-Borgen and Torstveit found subclinical or clinical eating disorders in 24% of their female and 9% of their male endurance athletes, respectively [4]. The percentages at risk for screening positive were higher in our collegiate distance running population than what these studies found in both the non-athlete and athlete population; however, due to different methods and study tools, it is difficult to provide direct comparison of these numbers. While some studies show higher rates of eating disorders in athletes [3, 4], others do not find a difference [1, 2]. As this study does not have a population of non-athletes or athletes from other sports screened with the ESP, it remains unclear how collegiate distance runners would compare to these groups. Thus, this is an area for further study.

Overall, the results highlight the high risk of eating disorders in collegiate distance runners. Comparing studies on eating disorder prevalence in athletic populations is challenging, as studies differ in populations with regard to sports, competition levels, and diagnostic tools. Nevertheless, this study, like others, brings to focus the impact of eating disorders in one subset of college athletes, namely distance runners. It emphasizes the high risk of disordered eating in this unique population, highlighting a need for these athletes to be studied separately from those of other sports for both males and females. Unfortunately, aggregate studies in male and female athletes will not appropriately reflect results for distance runners, as the perceived performance enhancement with weight varies between sports. That is, there are different ideal physiques and psychological stressors between not only genders but also sports [12], and eating disorder rates and risk are likely to be affected by these [9, 19, 20].

Several opportunities for future research evolved from this study. Perhaps most importantly, we encourage future research to employ alternative survey techniques in order to test the robustness of our findings, as we recognize that the ESP is only one method for screening for eating disorders. Future research should also examine the prevalence of disordered eating among other athletic populations. For instance, some sports emphasize bulk, whereas others promote a disordered eating style of binging and purging [12]. Thus, future studies would also benefit from examining variation in the risk of eating disorders by factors such as weight or BMI. Moreover, although we are unable to fully disentangle which event the participants competed in most frequently, future research should consider whether there is any variation in the risk of eating disorders between men and women across distances (i.e., a comparison between 800 meter runners to 5000 meter runners).

In addition to sport and distance variants, as well as pressures on body composition, future research should examine the potential mechanisms that explain the gender differences in eating disorder among distance runners. For instance, depression and low self-esteem are psychological factors that may contribute to the development of eating disorders [6]. Coach and peer relationships have also been noted to have both positive and negative impact on eating disorders, respectively, based on the nature of the interactions [21]. Moreover, training differences and approaches to running between cultures may also have an impact, as it has been shown that characteristics of the female athlete triad are not as prevalent in some African countries [22].

Another important element of this study is that future research cannot be limited solely to females, as 14% of males were at risk for screening positive for eating disorders. This suggests that disordered eating may have significant consequences on male student-athletes, at least those participating in distance running sports such as cross country and track. Indeed, a large proportion of the positive screens may have an eating disorder or subclinical eating disorders that would be diagnosed on further testing. The high percentage of positive screens is alarming considering that a recent study published in 2016 showed less than half of US collegiate sports medicine departments perform written or verbal screening for eating disorders, with Division I institutions having only a slightly higher screening rate at 52.5% [23]. Results were consistent with a previous study from 2003 showing only 60% of athletic departments screened for eating disorders, with only 6% using validated screening tools [24].

Additionally, athletic trainers—i.e., the members of the sports medicine team with the closest contact with the athletes—are not adequately equipped to identify eating disorders without the assistance of these screens. A recent study showed that only 68.1% of certified athletic trainers for colleges had ever attended an education program on disordered eating, and of these, only 28% had done so within the year [25]. A fundamental problem lies in the fact that athletic trainers do not feel as though they receive enough education on working with athletes with eating disorders [26]. A 2018 study found that while almost all athletic trainers working at NCAA institutions have heard of the Female Athlete Triad, only 32.98% had heard of Relative Energy Deficiency in Sport (RED-S). Additionally, most athletic trainers were only able to identify two components of the Female Athlete Triad highlighting a continued need for education on this topic [27]. Indeed, there should be great concern that many student athletes are suffering from eating disorders and their disease is being overlooked.

While the study had a larger number of responses from male student-athletes (366) compared to female student-athletes (272), this does not limit the overall impact of this study in highlighting the concern for eating disorders in collegiate distance runners. In fact, it may lead to an underestimate of the risk. Based on the study design, the response rate is unknown as athletes were contacted through their coaches. This creates a limitation in the study and possible selection bias. It is possible there was a lower response rate amongst either the female or male group. It could be hypothesized that those with eating disorders or concerns about their weight would be less likely to respond, possibly leading to an underreporting of the number of positive screens for eating disorders. Also, the coaches who forwarded the survey to their athletes may have a different team culture than coaches who did not reply.

An additional limitation stems from the number of schools who participated in the survey. While an attempt was made to email all Division I track and cross country coaches, only coaches from 25 men's programs and 25 women's programs responded that they forwarded the survey to their athletes. An additional three coaches responded that they would not forward the survey to their athletes, with 2 citing university policies. The remaining NCAA coaches either did not receive the email or did not respond. This does create another potential selection bias in this study as well as possible sampling error; and thus, we caution against interpreting our results as representative of the entire NCAA distance running population. We encourage future research to further examine these questions with alternative sources of data.

Other limitations include the screening tool used for eating disorders (i.e., the ESP). While the ESP may not be diagnostic of eating disorders or as frequently used as the 26 question “Eating Attitudes Test” or 50 question “Questionnaire for Eating Disorder Diagnosis,” it has been found to be comparable in several studies and noted by International Olympic Committee as a screening tool in athletes. Additionally, its brevity and less intrusive question style may have led to a better response from the student athletes.

## 5. Conclusions

The results of this study highlight the risk of eating disorders in NCAA Division I distance runners participating in cross country and track. While further data are needed to ascertain the true prevalence of eating disorders in the population, the high sensitivity and specificity associated with the ESP suggest that these results have highlighted an important issue of eating disorders among distance running college athletes. And although this study is limited in its implementation of only one screening tool for eating disorders, we believe that this does not preclude the need for implementation of procedures and policies in college athletics to ensure proper screening of student-athletes. Based on these results, it is imperative for all sports medicine and athletic departments to ensure appropriate screening for eating disorders. The authors would also recommend the implementation of evidence-based prevention programs for student-athlete populations with high numbers of disordered eating, such as distance runners, at institutions where resources are available.

## Figures and Tables

**Table 1:** Positive response rates to individual questions in the Eating Disorder Screen for Primary Care (ESP) by male and female cross country and track student athletes.

	Male		Female
Total Respondents	366		272

Are you dissatisfied with your eating patterns?	18.85% ± 2.05	*∗∗*	30.51% ± 2.80
Do you ever eat in secret?	7.92% ± 1.41	*∗∗∗*	27.94% ± 2.73
Does your weight affect the way you feel about yourself?	28.69% ± 2.37	*∗∗∗*	69.49% ± 2.80
Do you currently suffer with or have you ever suffered in the past with an eating disorder?	6.28% ± 1.27	*∗∗∗*	27.21% ± 2.70

Average Count of Positive Responses	.62 ± .09	*∗∗∗*	1.55 ± .15

*∗p*<.05, *∗∗p*<.01, and *∗∗∗p*<.001.

**Table 2:** Screening rates for Eating Disorders in NCAA Division I male and female cross country and track student-athletes using the Eating Disorder Screen for Primary Care (ESP).

	Male		Female
Average Age	19.99 ± .07		19.86 ± .08
Average BMI	20.81 ± .08	*∗*	20.44 ± .12
*Negative Screen*	86.34% ± 1.80	*∗∗∗*	54.05% ± 3.03
0 Positive Responses	57.65% ± 2.59	*∗∗∗*	22.06% ± 2.52
1 Positive Response	28.69% ± 2.37		31.99% ± 2.83
*Positive Screen*	13.66% ± 1.80	*∗∗∗*	45.95% ± 3.03
2 Positive Responses	9.02% ± 1.50	*∗∗∗*	24.26% ± 2.60
3 or 4 Positive Responses	4.64% ± 1.10	*∗∗∗*	21.69% ± 2.50

*∗p*<.05, *∗∗p*<.01, and *∗∗∗p*<.001.

## Data Availability

The data used to support the findings of this study are available from the corresponding author upon request.
